# Hyperoside Protected Against Oxidative Stress-Induced Liver Injury *via* the PHLPP2-AKT-GSK-3β Signaling Pathway *In Vivo* and *In Vitro*

**DOI:** 10.3389/fphar.2020.01065

**Published:** 2020-07-17

**Authors:** Haiyan Xing, Ruoqiu Fu, Caiyi Cheng, Yongqing Cai, Xianfeng Wang, Dongmei Deng, Xiaoyuan Gong, Jianhong Chen

**Affiliations:** ^1^Department of Pharmacy, Daping Hospital, Army Medical University, Chongqing, China; ^2^Center for Joint Surgery, Southwest Hospital, Army Medical University, Chongqing, China

**Keywords:** hyperoside, liver injury, oxidative stress, pleckstrin homology domain leucine-rich repeat protein phosphatase 2, nuclear factor erythroid-2-related factor 2, glycogen synthase kinase 3β

## Abstract

Hyperoside, isolated from *Drosera rotundifolia* L., seeds of *Cuscuta chinensis* Lam., or *Hypericum perforatum* L., originally showed to possess an antifungal and antibacterial activity, while recently showed the protective effects against oxidative stress-induced liver injury. This study investigated such a protective effect of hyperoside and the underlying molecular mechanisms *in vitro* and in carbon tetrachloride (CCl4)-injured rat livers. The data showed that hyperoside was able to prevent the oxidative stress-induced liver morphological changes and CCl4-induced rat liver injury. Hyperoside reversed the decrease of superoxidase dismutase (SOD) level and the increase of malondialdehyde (MDA) level *in vivo*. Moreover, hyperoside regulated the pleckstrin homology (PH) domain leucine-rich repeat protein phosphatase 2 (PHLPP2)-protein kinase B (AKT)-glycogen synthase kinase 3β (GSK-3β) signaling pathway in tert-butylhydroquinone (t-BHP)-treated liver cells, e.g., Hyperoside reduced PHLPP2 expression to activate AKT phosphorylation, induce GSK-3β phosphorylation, and then increased nuclear factor erythroid-2-related factor 2 (Nrf2) nuclear translocation, reduced nuclear translocation of phosphorylated Fyn, and promoted heme oxygenase-1 (HO-1) expression *in vivo* and *in vitro*. In contrast, siRNA-mediated knockdown of PHLPP2 expression enhanced hyperoside-mediated activation of the AKT-GSK-3β kinase pathway in liver cells. In conclusion, the present study demonstrated that hyperoside could protect against oxidative stress-induced liver injury by regulating the PHLPP2-AKT-GSK-3β signaling pathway *in vivo* and *in vitro*.

## Introduction

Oxidative stress contributes to the pathophysiological processes of alcoholic liver disease, non-alcoholic fatty liver disease, drug-induced liver disease, and other liver diseases by producing excessive reactive oxygen species (ROS), destroying mitochondrial structure and function, and reducing the level and activity of superoxide dismutase (SOD) and other cytoprotective proteins (Ivanov et al., 2017). Therefore, hepatic ROS is thought to be central in the pathogenesis of liver injury, and antioxidants including vitamin C and phenolic compounds have been extensively investigated to determine their ability to confer liver protection (Chougule and Kekunnaya, 2019; Fan et al., 2019; Su et al., 2019).

Natural products, especially phytochemicals, have attracted our attention in drug discovery and food supplement research (Zhang et al., 2019). Hyperoside (Hyp), isolated from *Drosera rotundifolia* L., seeds of *Cuscuta chinensis* Lam., or *Hypericum perforatum* L. originally showed to possess numerous pharmacological effects, including anti-inflammatory, anti-thrombotic, anti-diabetic, anti-viral, anti-fungal, hepato-protective (Choi et al., 2011), and especially anti-oxidative properties (Li et al., 2005; Park et al., 2016). *More recently*, Hyp exhibits an anti-oxidative activity against a series of diseases or conditions, such as carbon tetrachloride (CCl4)-induced liver injury (Zou et al., 2017), and hepatic and renal ischemia-reperfusion injury (Shi et al., 2019; Wu et al., 2019). However, the exact mechanisms involved in Hyp-mediated protection of the oxidative damaged liver are not completely understood.

The endogenous anti-oxidant system is composed of heme oxygenase-1 (HO-1), superoxide dismutase (SOD), glutathione (GSH), NQO1, and other cytoprotective proteins. The upstream promoter regions of these protein-coding genes contain an anti-oxidant response element (ARE), whose transcription is regulated by the transcription factor nuclear factor erythroid-2-related factor 2 (Nrf2). When oxidative stress or anti-oxidant induction signals occur, Nrf2 rapidly translocates from the cytoplasm to the nucleus where it is activated. Nrf2 then forms a heterodimer with small musculoaponeurotic fibrosarcoma protein, which binds to the GCTGAGTCA site on the ARE sequence of the cell protection gene. Interestingly, recent studies have found that pleckstrin homology domain leucine-rich repeat protein phosphatase (PHLPP) can dephosphorylate AKT (Ser473), while AKT can phosphorylate and inhibit GSK-3β activity (Rajala et al., 2016), and therefore indirectly and positively regulate GSK-3β. GSK-3β can also mediate phosphorylation of PHLPP through a feedback mechanism, leading to ubiquitination and protein degradation by beta-TrCP (Li et al., 2009).

The PHLPP family comprises of two members of serine/threonine phosphatases (PHLPP1 and PHLPP2). It is reported that PHLPP1 is most highly expressed in the brain and heart (Miyamoto et al., 2010; Shimizu et al., 2010), while down-regulation of PHLPP2 is closely associated with liver protection (Rizvi et al., 2015).We previously reported that Hyp can induce phosphorylation of GSK-3β at Ser9 without affecting GSK-3β expression and its phosphorylation at Thr390 (Xing et al., 2015). However, it remains unclear if Hyp-mediated GSK-3β inactivation involves PHLPP2. In this study, we designed both *in vivo* and *in vitro* experiments to determine the role of the PHLPP2-AKT-GSK-3β signaling pathway on the protective effects of Hyp against oxidative stress-induced liver injury using pharmacological and genetic approaches. This study provides important evidence supporting the anti-oxidant effects and mechanisms of Hyp to protect against liver damage.

## Materials and Methods

### Antibodies and Reagents

Hyp with a purity of ≥ 98% was purchased from Nanjing Zelang Medical Technological Co., Ltd (Nanjing, China), while CCl4 and tert-butylhydroquinone (t-BHQ) were obtained from Sigma Chemical (St. Louis, MO, USA). Moreover, rabbit antibodies anti-PHLPP2 (Cat. #SAB1300919; Sigma), anti-AKT (Cat. #9272; Cell Signaling Technology, Danvers, MA, USA), anti-phosphorylated (p)-AKT (S473) (Cat. #4060; Cell Signaling Technology), anti-p-AKT (Thr308) (Cat. #2965; Cell Signaling Technology), anti-GSK-3β (clone #3D10; Cat. #12456; Cell Signaling Technology), anti-p-GSK-3β (Ser9) (Cat. #9322; Cell Signaling Technology), anti-Fyn (Cat. #4023; Cell Signaling Technology), anti-Histone H2 (Cat. #2595; Cell Signaling Technology), anti-Nrf2 (pS40) (Cat. #ab89443; Abcam, Cambridge, MA, USA), and anti-Nrf2 (Cat. #ab89443, Abcam), and anti-HO-1 (Cat. #SC-10789; Santa Cruz Biotechnology, Santa Cruz, CA, USA), anti-p-Fyn (Thr) (Cat. #sc-5267; Santa Cruz Biotechnology), and a mouse antibody anti-GAPDH (Cat. #SC-365062; Santa Cruz Biotechnology) were purchased from the named companies, respectively.

### Animals and Experiments

The animal protocol of this study was approved by the Institutional Animal Care and Use Committee (IACUC) of the Army Medical University (Chongqing, China) (Approval #201510003 and 201612010). All animal experiments followed the ARRIVE guidelines and were carried out in accordance with the National Institutes of Health guide for the care and use of Laboratory animals (NIH Publications No. 8023, revised 1978). Male 2-month-old Sprague-Dawley rats with 200 ± 20 g body weight were purchased from the Experimental Animal Center of The Army Medical University. Animals were kept in Specific pathogen free (SPF) facility and fed a standard laboratory diet with free access to water in temperature-controlled room (22 ± 1°C) with a humidity of 65 ± 5% at a 12:12 h light/dark cycle. For our experiments, these rats were divided into seven groups (n = 6 in each group), i.e., the control, CCl4, high dose Hyp (60 mg/kg) + CCl4, moderate dose Hyp (30 mg/kg) + CCl4, low dose Hyp (15 mg/kg) + CCl4, high dose Hyp-only (60 mg/kg), and positive control of morin (30 mg/kg) + CCl4 group. The CCl4 was dissolved in corn oil at the 1:1 ratio and intraperitoneally injected into the rats at a dose of 1.5 ml/kg to produce acute liver injury. After adaption for 1 week, the rats were orally administrated different doses of Hyp (dissolved in saline at amount of 0.8 ml/100 g) for 3 consecutive days, while the control rats received the same volume of saline. The rats were then intraperitoneally injected with CCl4 6 h after the last drug treatment. After 16 h, blood was collected from the heart following anesthesia with isoflurane (5% for induction and 2% for maintenance). The animals were then decapitated and liver tissues were collected for subsequent experiments.

### Histological Analysis

Liver tissues resected from the rats were fixed in 4% paraformaldehyde for 48 h and processed for paraffin embedding and preparation of 5-µm consecutive sections. The tissue sections were stained with hematoxylin and eosin (HE) and revised for morphological changes under a light microscope (BX51, Olympus) by two researchers in a blinded manner to the experimental groups.

### Biochemical Assays

Serum alanine aminotransferase (ALT) and aspartate transaminase (AST) were measured using the assay kits from Nanjing Jiancheng Bioengineering Institute (Cat. #C009-2 and #C010-2, respectively; Nanjing, China), while the liver malondialdehyde (MDA) and SOD levels were assayed using the assay kits from Nanjing Jiancheng Bioengineering Institute (Cat. #A003-1 and #A001-3 respectively; Nanjing, China) in liver tissues according to the manufacturers’ protocols. Specifically, the sera from the rats were used according to the dilution of the kits, while the liver tissues were lysed using a radioimmunoprecipitation assay buffer (RIPA buffer) and centrifuged at 3,000 rpm at 4°C for 10 min to collect the supernatants.

### Cell Culture and Treatment

Human hepatocytes (L02 cells) were obtained from the Cell Bank Type Culture Collection of the Chinese Academy of Sciences (Shanghai, China) and cultured in HyClone RPMI1640 medium (Thermo Scientific, Beijing, China) supplemented with 10% (v/v) fetal bovine serum (FBS) (Gibco, USA), 100 U/ml penicillin, and 100 μg/ml streptomycin at 37°C in a humidified atmosphere at 5% CO_2_. Cells of 3–5 passages were used in our study. Hyp was dissolved in dimethylsulfoxide (DMSO) as a stock solution and diluted with the culture medium before cell culture treatment, while control cells were treated with equal amounts of DMSO at a final concentration of < 0.1%.

The dose of Hyp and morin was selected according to previous publications (Zhu et al., 2012; Madankumar et al., 2014; Li et al., 2018). To assess the effects of Hyp on expression of PHLPP and related proteins, liver cells were treated with morin (10 μM for 3 h) or Hyp (100 μM for 1, 3, or 6 h), while to assay the effect of Hyp on t-BHP-induced protein expression, cells were treated with morin (10 μM) and Hyp (100 μM) for 3 h and 6 h, respectively and then with t-BHP (250 μM for 90 min). Thereafter, the cells were collected for the following experiments.

### Western Blot

To detect levels of HO-1, Nrf2, AKT, p-AKT, PHLPP2, Fyn, GSK-3β, p-Fyn, and p-GSK-3β, we prepared nuclear, cytoplasmic proteins, or total cellular protein using the Nuclear and Cytoplasmic Protein Extraction Kit (Cat. #P0028, Beyotime, Shanghai, China) according to a previous study (Xing et al., 2015). For preparation of nuclear extracts, the cells were gently removed by mechanical scraping and collected by centrifugation at 1,500 g for 5 min. The cell pellet was resuspended in 5 ml of cell lysis buffer [10 mM HEPES (pH 7.9), 1.5 mM MgCl_2_, 10 mM KCl, 0.5 mM dithiothreitol (DTT) and 0.2 mM phenylmethylsulfonyl fluoride (PMSF)[, and immediately centrifuged at 1,500 g for 5 min. Nuclei were collected by centrifugation at 3,300 g for 15 min at 4°C. The nuclei were resuspended in nuclear extraction buffer [20 mM HEPES (pH 7.9), 1.5 mM MgCl_2_, 400 mM KCl, 0.5 mM DTT, 0.2 mM PMSF, and 25% glycerol]. The supernatant was collected by centrifugation at 14,000 g for 3 h at 4°C. For preparation of cytosolic extracts, the supernatant obtained after removal of nuclei was mixed thoroughly with 0.11 volume of 10× cytoplasmic extraction buffer [1×: 30 mM HEPES (pH 7.9) at 4°C, 140 mM KCl, 3 mM MgCl_2_] and then centrifuged at 89,000 g for 1 h. The supernatant was collected and concentrated by centrifugation at 14,000 g for 1 h at 4°C. The protein concentrations were determined using the bicinchoninic acid protein assay kit (Pierce, Rockford, IL, USA). The levels of p-Nrf2 and Fyn were analyzed in both nuclear and cytoplasmic fractions of cell lyses, while total protein was used to analyze other proteins.

For western blot analysis, equal amounts of protein samples (30 μg each loading) were separated using 10% sodium dodecyl sulfate-polyacrylamide gel electrophoresis (SDS-PAGE) (Bio-Rad Laboratories, Inc., Hercules, CA, USA) and transferred onto the nitrocellulose membranes (Pierce, Rockford, IL, USA). The membranes were then blocked in a non-fat dried milk solution (5% in Tris-buffered saline) at the room temperature for 2 h and incubated with appropriate primary antibodies at 4°C overnight according to the manufacturers’ instructions. On the next day, the membranes were briefly washed with TBS-Tween 20 (TBS-T) thrice and incubated with the secondary antibodies and stained with the enhanced chemiluminescent reagents (Thermo Scientific, Beijing, China) for detection of positive protein signaling. The Histone H2 and GAPDH were used as references for nuclear extracts and total/cytosolic extracts, respectively. The western blots were then quantified by using Image J Software (National Institute of Heath, Bethesda, MD, USA).

### Short Interfering RNA (siRNA) and Cell Transfection

Three PHLPP siRNAs and one negative control siRNA were designed and synthesized by Shanghai Gene Pharma Co., Ltd (Shanghai, China). For cell transduction, the siRNA solution was mixed with respective volumes of Lipofectamine 2000 (Invitrogen, Carlsbad, CA, USA) for 20 min to form siRNA liposomes and then transfected into L02 cells in antibiotic-free cell culture medium for 48 h before Hyp or t-BHQ treatment.

### Quantitative Reverse Transcriptase-Polymerase Chain Reaction (qRT-PCR)

Total cellular RNA was isolated from treated cells or liver tissues using the Trizol reagent (Invitrogen) and 2.0 μg of each RNA samples were reversely transcribed into cDNA using the Transcriptor First Strand cDNA Synthesis Kit (Roche, Indianapolis, IN, USA) according to the manufacturer’ instruction. These cDNA samples were then amplified using qPCR with primers and the SYBR Green dye/the TaqMan Master Mix (Applied Biosystems, Foster City, CA, USA) in a 7900 Real Time System (Applied Biosystems). The qPCR conditions were 94°C for 30 s, 59°C for 30 s, and 72°C for 45 s for 40 cycles. The primers were synthesized by Shanghai Sangon Biological Engineering Technology & Services (Shanghai, China) and the primer sequences were PHLPP2, 5’-CAATGAGCAAGGACAGGAT-3’ and 5’-GGTCCTCTGGTTCCATCTGA-3’ and GAPDH, 5’-CAATGACCCCTTCATTGACC-3’ and 5’-GACAAGCTTCCCGTTCTCAG-3’. PHLPP2 level was normalized to GAPDH mRNA and quantified using the 2-^ΔΔ^Ct method (Livak and Schmittgen, 2001; Zhu et al., 2015).

### Statistical Analysis

All data were expressed as means ± standard deviation (SD) and analyzed using one-way analysis of variance (ANOVA) followed by the SNK-q test for multiple comparisons with the Statistical Package for the Social Sciences (SPSS 20) software (Chicago, IL, USA). A P-value < 0.05 as considered statistically significant.

## Results

### Hyp Protection Against CCl4-Induced Rat Liver Injury

**Figure 1:**
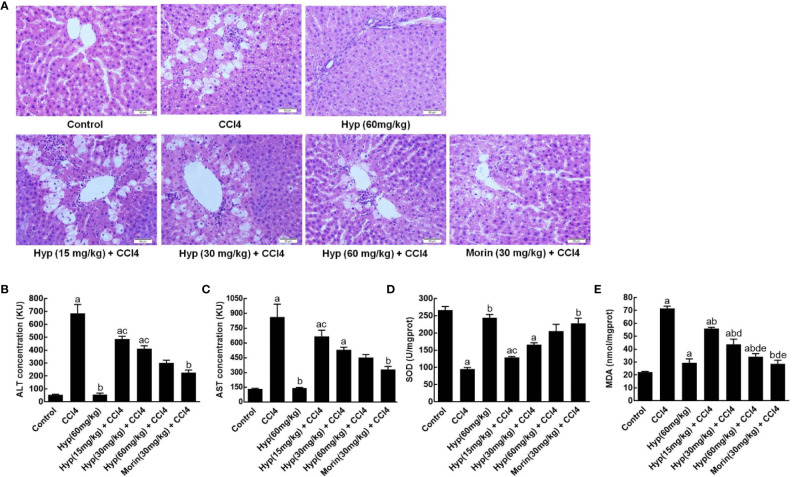
Hyperoside protection of CCl4-induced rat liver morphological changes and injury. **(A)** HE staining of liver tissues. Morphological analysis of liver tissues showed that typical liver injury, including edema, nuclear condensation, and vacuolization, was evident in the model group. By contrast, hyperoside dose-dependently prevented the injury at the range from 15 mg/kg to 60 mg/kg. **(B)** ALT levels. **(C)** AST levels. **(D)** SOD levels. **(E)** MDA levels. All data are expressed as mean and standard deviation (SD) and statistically analyzed by using the ANOVA. ^a^P < 0.05 vs. Control, ^b^P < 0.05 vs. CCl4, ^c^P < 0.05 vs. Hyp (60 mg/kg), ^d^P < 0.05 vs. Hyp (15 mg/kg + CCl4), and ^e^P < 0.05 vs. Hyp (30 mg/kg + CCl4).

In this study, we first established CCl4-induced liver injury in rats and found morphological changes in rat liver tissues after CCl4 injections, including liver cell edema, nuclear condensation, and vacuolization. However, treatment with Hyp at doses between 15 and 60 mg/kg was able to protect the liver tissues from CCl4-induced injury (**Figure 1A**). Biochemically, Hyp treatments also protect the rats from changes in the liver enzymes, i.e., the ALT and AST levels significantly increased in the model group, but Hyp treatment reversed their changes (**Figures 1B, C**). Furthermore, we also measured the oxidative status of the liver tissues and found that SOD level was decreased, and MDA level was increased in the model group, whereas Hyp treatment reversed these SOD and MDA levels in the liver tissues (**Figures 1D, E**).

### Hyp Protection of CCl4-Induced Liver Oxidative Stress by Regulation of HO-1, Fyn, and Nrf2 Expression

**Figure 2:**
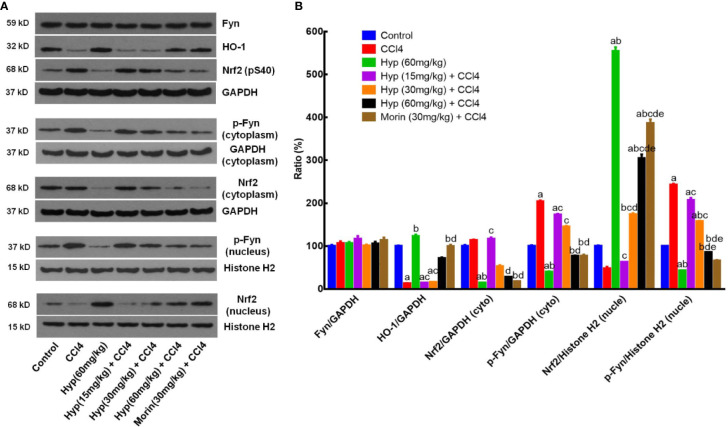
Hyperoside protection of CCl4-induced liver injury against oxidative damages *in vivo*. **(A)** Western blot. The expressions of total Fyn, HO-1, p-Nrf2 (S40), the cytoplasmic and nuclear p-Fyn and Nrf2 were analyzed respectively in liver tissues from animal experiments. **(B)** Representative quantification data of Fyn, HO-1, cytoplasmic Nrf2, cytoplasmic p-Fyn, nuclear Nrf2 and nuclear p-Fyn. These western blots were quantified by using Image J Software and the data are expressed as mean and standard deviation (SD) and statistically analyzed by using the ANOVA. ^a^P < 0.05 vs. Control, ^b^P < 0.05 vs. CCl4, ^c^P < 0.05 vs. Hyp (60 mg/kg), ^d^P < 0.05 vs. Hyp (15 mg/kg + CCl4), and ^e^P < 0.05 vs. Hyp (30 mg/kg + CCl4).

HO-1, Fyn, and Nrf2 are important factors in cell oxidative stress (Biswas et al., 2014) and we, therefore, detected expression of these proteins in CCl4-injured liver. We found that CCl4 injection reduced HO-1 expression, but induced p-Nrf2 level, both cytoplasmic and nuclear p-Fyn expressions in the liver tissues (**Figure 2**), whereas Hyp treatment (60 mg/kg) reversed these changes in the liver tissues (P < 0.05 vs. control or vs. CCl4). Hyp treatment also induced nuclear Nrf2 expression (P < 0.05 vs. control or vs. CCl4), reduced cytoplasmic Nrf2, but the total Fyn levels in each group were comparable (**Figure 2**).

### Hyp Regulation of the PHLPP-AKT-GSK-3β Signaling Pathway in t-BHP-Treated Liver Cells

**Figure 3:**
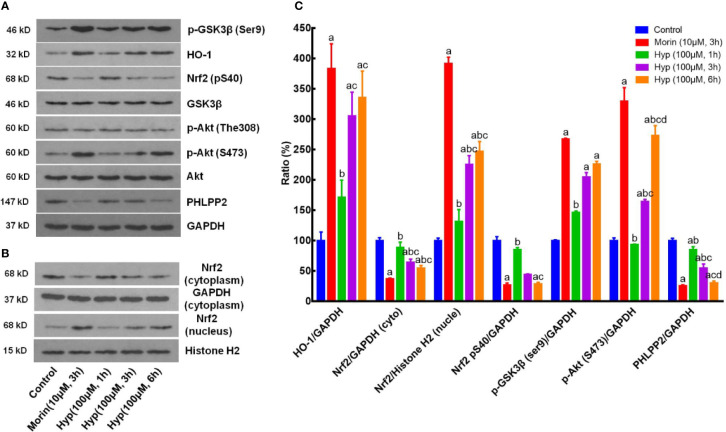
Hyperoside regulation of the PHLPP2-AKT-GSK-3β signaling pathway in liver L02 cells. **(A)** Western blots. The cytoplasmic HO-1, p-GSK-3β, p-AKT, and PHLPP2 were analyzed using western blotting in liver L02 cells after treated with t-BHP. **(B)** Representative blots of cytoplasmic and nuclear Nrf2. **(C)** Quantification data of HO-1, cytoplasmic Nrf2, nuclear Nrf2, p-Nrf2 (S40), p-GSK-3β, p-AKT (S473), and PHLPP2. These western blots were quantified by using Image J Software and the data are expressed as mean and standard deviation (SD) and statistically analyzed by ANOVA. ^a^P < 0.05 vs. Control, ^b^P < 0.05 vs. Morin, ^c^P < 0.05 vs. Hyp for 1 h, and ^d^P < 0.05 vs. Hyp for 3 h.

We next sought to confirm our *in vivo* data in cultured liver cells by treating with t-BHP to mimick the oxidative stress conditions. We found that Hyp (100 μM) induced expression of p-AKT, p-GSK-3β, HO-1, and nuclear Nrf2, but reduced expression of cytoplasmic Nrf2, p-Nrf2, and PHLPP2 in liver cells at both 3 and 6 h treatment in a time-dependent manner (**Figure 3**).

**Figure 4:**
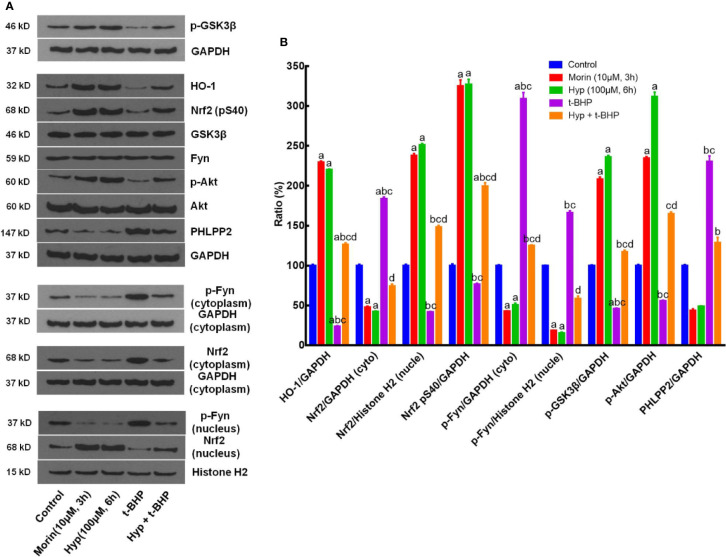
Hyperoside regulation of the PHLPP2-AKT-GSK-3β signaling pathway in t-BHP-treated liver L02 cells. **(A)** Western blots. The cytoplasmic and nuclear HO-1, Nrf2, p-GSK-3β, p-AKT, and PHLPP2 levels were assessed using western blotting in t-BHP-treated liver L02 cells. **(B)** Representative quantification data of HO-1, cytoplasmic Nrf2, nuclear Nrf2, p-Nrf2(S40), cytoplasmic p-Fyn, nuclear p-Fyn, p-GSK-3β, p-AKT, and PHLPP2. These Western blots were quantified by using Image J Software and the data are expressed as mean and standard deviation (SD) and statistically analyzed by ANOVA. ^a^P < 0.05 vs. Control, ^b^P < 0.05 vs. Morin, ^c^P < 0.05 vs. Hyp for 6 h, and ^d^P < 0.05 vs. t-BHP.

Moreover, we also evaluated the effects of Hyp on regulation of the PHLPP2-AKT-GSK-3β signaling pathway in the t-BHP-induced L02 cells. Morin, which had been reported to regulate Nrf2 stability by modulating PHLPP2 (Rizvi et al., 2015), was used as a positive control. Our data showed that t-BHP reduced HO-1 expression but enhanced cytoplasmic Nrf2 (P < 0.05 vs. the control), whereas Hyp induced expression of p-AKT, p-GSK-3β, nuclear Nrf2, p-Nrf2(S40), and HO-1 (P < 0.05 vs. t-BHP), but reduced cytoplasmic Nrf2, p-Fyn, and PHLPP2 expression, while total Fyn level was not affected by Hyp treatment (**Figure 4**).

**Figure 5:**
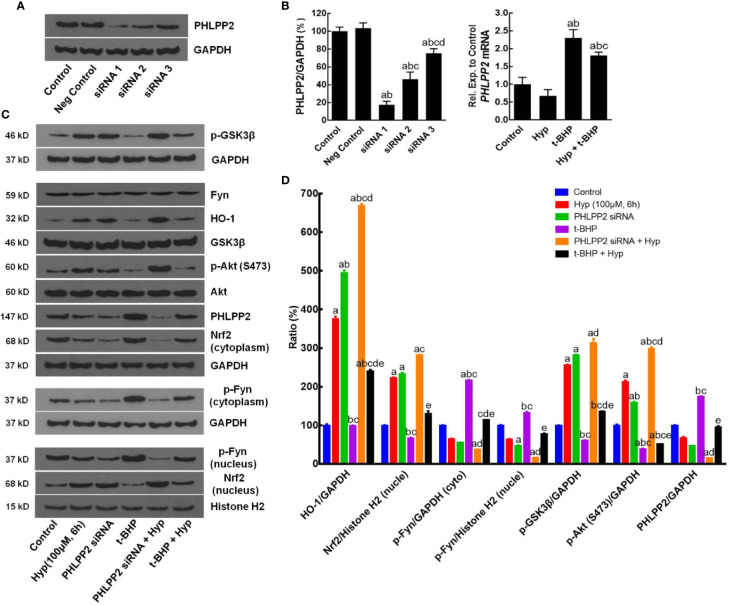
Enhancement of Hyperoside-regulated PHLPP2-AKT-GSK-3β signaling pathway after knockdown of PHLPP2 expression in liver L02 cells. **(A)** Western blots. Levels of these different proteins were analyzed using western blotting in L02 liver cells after PHLPP2 siRNA1 transfection with or without t-BHP treatment. **(B)** Quantified data on PHLPP2 expression after PHLPP2 siRNA transfection (Left: protein expression; Right: mRNA expression). ^a^P < 0.05 vs. the control, ^b^P < 0.05 vs. the negative control, ^c^P < 0.05 vs. PHLPP2 siRNA1, ^d^P < 0.05 vs. PHLPP2 siRNA2. **(C)** Representative blots. **(D)** Representative quantification data on HO-1, nuclear Nrf2, cytoplasmic p-Fyn, nuclear p-Fyn, p-GSK-3β, p-AKT, and PHLPP2. These western blots were quantified by using Image J Software and the data are expressed as mean and standard deviation (SD) and statistically analyzed by ANOVA. ^a^P < 0.05 vs. Control, ^b^P < 0.05 vs. Hyp for 6 h, ^c^P < 0.05 vs. PHLPP2 siRNA, ^d^P < 0.05 vs. the t-BHP treatment, and ^e^P < 0.05 vs. PHLPP2 siRNA + Hyp.

After that, we assessed the role of PHLPP2 in the regulation Nrf2 signaling pathway by using PHLPP2 siRNAs to knockdown PHLPP2 expression in L02 cells. We found that compared with that of the negative control, PHLPP2 siRNAs remarkably reduced PHLPP2 expression in liver cells (**Figures 5A, B**), especially PHLPP2 siRNA1; thus, it was selected for our subsequent experiments. Indeed, PHLPP2 siRNA1 transfection induced expression of HO-1, nuclear Nrf2, and p-AKT, but reduced expression of cytoplasmic Nrf2 and p-Fyn in control cells (P < 0.05 vs. the control). PHLPP2 siRNAs also reduced the t-BHP-induced increase in cytoplasmic Nrf2, p-Fyn and PHLPP2 expression, and reversed the decreased levels of HO-1, nuclear Nrf2, p-AKT (S473), and p-GSK-3β (P < 0.05 vs. t-BHP; **Figures 5C, D**).

### Hyp Regulation of the PHLPP2-AKT-GSK-3β Signaling Pathway in CCl4-Injured Rat Liver Tissues

**Figure 6:**
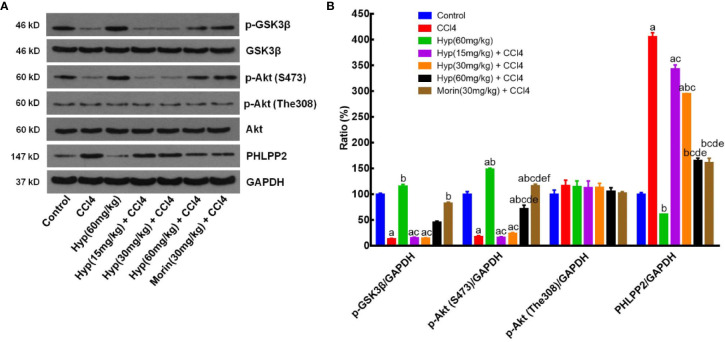
Hyperoside regulation of the PHLPP2-AKT-GSK-3β signaling pathway in CCl4-injured rat liver. **(A)** Western blots. Levels of cytoplasmic and nuclear p-GSK-3β and p-AKT proteins were assessed using western blotting in CCl4-injured rat liver tissues. **(B)** Representative quantification data on p-GSK-3β, p-AKT (S473), p-AKT (The308), and PHLPP2. These western blots were quantified by using Image J Software and the data are expressed as mean and standard deviation (SD) and statistically analyzed by the ANOVA. ^a^P < 0.05 vs. Control, ^b^P < 0.05 vs. CCl4, ^c^P < 0.05 vs. Hyp (60 mg/kg), ^d^P < 0.05 vs. Hyp(15 mg/kg + CCl4), ^e^P < 0.05 vs. Hyp(30 mg/kg + CCl4), and ^f^P < 0.05 vs. Hyp(60 mg/kg + CCl4).

After that, we further assessed the changed expression of PHLPP2, AKT, and GSK-3β proteins in CCl4-injured rat liver tissues. As shown in **Figure 6**, levels of total AKT and GSK-3β proteins in each group were comparable, but levels of p-AKT and p-GSK-3β levels were significantly reduced in the CCl4 group (vs. the control). However, Hyp (60 mg/kg) treatment reversed the CCl4-induced decrease in p-AKT expression, while Hyp also promoted p-GSK-3β (P < 0.05 vs. CCl4). CCl4 injection induced PHLPP2 expression (P < 0.05 vs. the control), but treatment with Hyp (30 and 60 mg/kg) reversed CCl4-induced PHLPP2 expression in rat liver tissues (**Figure 6**).

## Discussion

**Figure 7:**
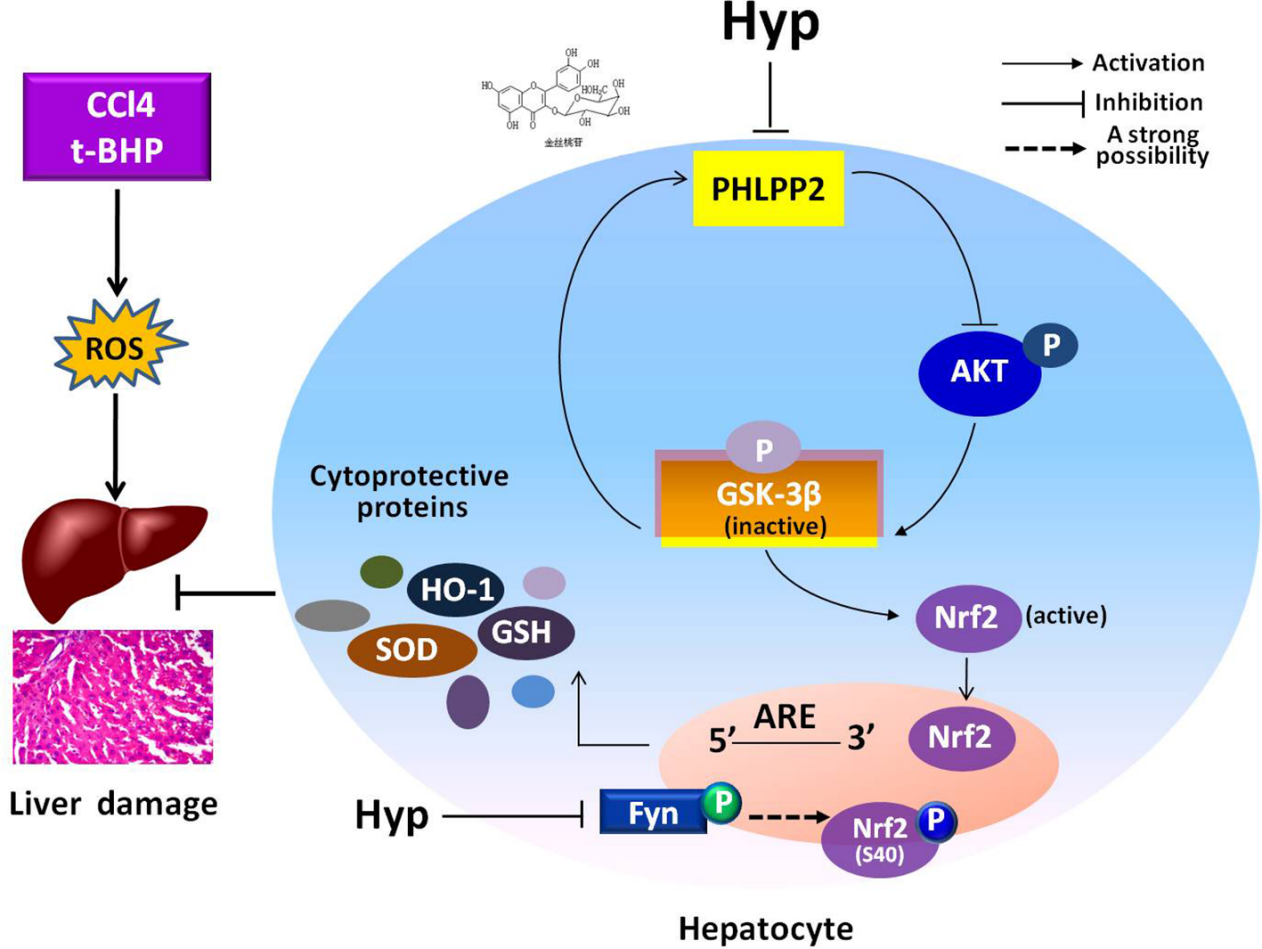
A schematic illustrating the mechanisms involved in the protection of hyperoside against oxidative liver stress. Hyperoside was able to down regulate PHLPP2 expression in liver cells treated with t-BHP and in CCl4-injured rat liver to induce AKT phosphorylation and inactivate GSK-3β (Ser9), and thereby, reduced Fyn phosphorylation, and nuclear translocation. The phosphorylation of Nrf2 at S40 site was probably involved in Hyp-mediated regulation of Fyn kinase, likely through nuclear translocation for HO-1 transcription and translation. The dashed line highlights that this is a strong suggestion and has not been completely proven with experiment.

In the current study, we revealed the protective effects of Hyp on liver L02 cells and CCl4-injured rat liver through regulation of the PHLPP2-AKT-GSK-3β signaling pathway, i.e., Hyp was able to down regulate PHLPP2 expression in liver cells treated with t-BHP and in CCl4-injured rat liver to induce AKT phosphorylation and inactivate GSK-3β (Ser9), and thereby, promoted Nrf2 nuclear translocation for HO-1 transcription and translation (**Figure 7**).

CCl4-induced rat liver injury is the frequently used animal model to mimic damages to liver cell morphology and structure as well as biochemical alterations (Hamid et al., 2017) as liver diseases do (Xu et al., 2003). This animal model is also frequently used to assess different agents or drugs in protection against liver damages (Al-Harbi et al., 2014; Hamid et al., 2017). Thus, in the current study, we utilized this animal model to assess the protective effects of Hyp *in vivo*. Similarly, t-BHP-treated liver cells are also routinely used as an oxidative stress model to evaluate the anti-oxidative ability of agents (Kucera et al., 2014; Liu et al., 2017). Hyp is a potent antioxidant derived from plants with a chemical structure of quercetin 3-O-β-D-galactoside and previous studies showed its variety of biological activities, including myocardial protection, antioxidative effect, and anti-inflammatory activities (Qi et al., 2020). Hyp has been used to treat different diseases and clinical conditions (Zhang et al., 2014; Gao et al., 2019). t-BHP and H_2_O_2_ are typical oxidants, which can directly cause oxidative damage of liver cells (Kucera et al., 2014). In our previous *in vitro* study, we demonstrated that Hyp was able to protect liver injury (Xing et al., 2015), while others showed the Hyp protective effects in ischemia injury (Shi et al., 2019), heart failure-induced liver fibrosis (Guo et al., 2019), and toxins-induced liver injury (Xie et al., 2016; Zou et al., 2017). Our current study further supports these findings of Hyp antioxidant effects. However, further study is needed to verify the development of Hyp as a hepatic protection drug. The aim of this present study is to investigate the function of Hyp on oxidative stress-induced liver injury. As the effects of morin on normal liver cells have been evaluated in previous study (Rizvi et al., 2015), we did not include all data about morin in this present study. We selected morin as a positive control in the treatment of CCl4-induced liver injury.

Indeed, the endogenous antioxidant system includes the HO-1, SOD, GSH, NQO1, and other cytoprotective proteins (Jung and Kwak, 2010). The upstream promoter regions of these genes contain an antioxidant response element (ARE) that is regulated by the nuclear factor erythroid-2-related factor 2 (Nrf2) (Raghunath et al., 2018). During activation of the oxidative stress or antioxidant induction signals, Nrf2 rapidly translocates from the cytoplasm to the nucleus to be activated by formation of the Nrf2 heterodimer with small Maf protein to bind to the ARE consent sequences of 5’-GCTGAGTCA-3’ in the cell protective genes (Li et al., 2008). Furthermore, a recent study reported that PHLPP was able to dephosphorylate AKT (Ser473) to in turn phosphorylate and inhibit the GSK-3β activity and therefore, indirectly and positively regulated GSK-3β activity [12]. However, GSK-3β was able to regulate PHLPP phosphorylation through a feedback mechanism, leading to ubiquitination, and protein degradation through beta-TrCP (Li et al., 2009). Our previous study revealed that Hyp was able to induce phosphorylation of GSK-3β at Ser9 but not affect total GSK-3β expression or GSK-3β phosphorylation at Thr390 (Xing et al., 2015). In the current study, we designed and conducted both *in vivo* and *in vitro* experiments to assess the PHLPP2-AKT-GSK-3β signaling pathway in mediation of Hyp-induced anti-oxidative effects in liver injury and try to explore the role of this feedback loop as the molecular mechanism.

A previous study reported that flavones can upregulate expression of the Nrf2-mediated HO-1 and other cytoprotective genes *via* activation of multiple kinase pathways to restore the hepatocytes redox homeostasis (Tu et al., 2019), while Hyp had a remarkable protective effect against liver injury, myocardial injury, and kidney injury, which was also likely through activation of the Nrf2/ARE pathway (Lee and Jeong, 2016; Liang et al., 2018; Qiu et al., 2018). In our previous study, we showed that Hyp attenuated the H_2_O_2-_induced liver cell damage by activation of the Nrf2-ARE signaling pathway and increased GSK-3β phosphorylation at Ser9 (Xing et al., 2015). In the current study, we demonstrated that Hyp protected against CCl4-induced liver injury *in vivo* and t-BHP-induced liver cell injury *in vitro*, both of which were through induction of HO-1 expression and SOD levels, but down-regulation of MDA levels. Moreover, Fyn is a Src kinase family member to act as a negative Nrf2 regulator (Okada, 2012). During a stressful condition, Nrf2 translocates into the nucleus to regulate gene transcription, whereas Fyn can phosphorylate Nrf2 at 568-tyrosine site, resulting in Nrf2 nuclear exportation and degradation (Kaspar and Jaiswal, 2011). Our current study using t-BHP and CCl4 to produce *in vitro* and *in vivo* liver oxidative stress revealed that Hyp induced Nrf2 nuclear translocation, which was likely through reduction of Fyn phosphorylation and nuclear translocation. Surprisingly, the phosphorylation of Nrf2 at S40 site was probably involved in Hyp-mediated regulation of Fyn kinase. However, we found that under the effect of t-BHP, the regulatory effect of Hyp on phosphorylation of Nrf2 at S40 in L02 cells was opposite to that in CCl4-injured rats. That discrepancy might be caused by the difference of *in vitro* and *in vivo* models. Interestingly, Hyp exerted similar function to balance the level of phosphorylation of Nrf2(S40). Therefore, the association between the phosphorylation of Nrf2(S40) and Fyn in Hyp-induced activation of the Nrf2/ARE pathway still deserves clarification.

Furthermore, our previous study showed that Hyp-induced GSK-3β phosphorylation at Ser9 without affecting the total protein GSK-3β levels or GSK-3β phosphorylation at Thr390 to protect against H_2_O_2_-induced liver L02 cell damages (Xing et al., 2015). In our current study, we demonstrated that Hyp was also able to induce GSK-3β phosphorylation and block the t-BHP-induced reduction of GSK-3β phosphorylation *in vitro*, which was likely through downregulation of PHLPP2 expression. Taken together, after Hyp administration, levels of both PHLPP2 mRNA and protein were reduced and as a phosphatase, PHLPP can directly dephosphorylate AKT (Gao et al., 2005), leading to Hyp induction of AKT phosphorylation at S473 and in turn GSK-3β phosphorylation. Furthermore, knockdown of PHLPP2 expression enhanced the protective effects of Hyp on liver cells *in vivo* and *in vivo*. In other words, PHLPP2-siRNA exerted a similar function as Hyp, indicating that PHLPP2 reduction exerted a similar effect as Hyp did (Warfel and Newton, 2012). Taken together with data from a previous study (Mathur et al., 2016), our current study supports a novel mechanism by which PHLPP2 involves in the effects of antioxidants, like Hyp, through the GSK-3β/Fyn/Nrf2 pathway.

In addition, PHLPP-induced AKT dephosphorylation at Ser473 resulted in a 90% reduction in the intrinsic AKT catalytic activity and AKT-related cell survival signaling pathways (Kapoor and Kakkar, 2012). Interestingly, GSK-3β is able to phosphorylate PHLPP at Ser847, leading to PHLPP-dependent ubiquitination and protein degradation by the E3 ligase beta-TrCP, as the PHLPP2-AKT-GSK-3β feedback regulatory loop (Li et al., 2009). As reported previously, morin was able to protect primary hepatocytes and rat livers from t-BHP-induced injury by inhibition of PHLPP2 and activation of the AKT/GSK-3β/Nrf2 pathway (Mathur et al., 2016). However, our current study showed that Hyp inactivated GSK-3β through GSK-3β phosphorylation and promoted the Nrf2 pathway. GSK-3β-mediated PHLPP2 phosphorylation resulted in induction of PHLPP degradation *via* the E3 ligase β-TrCP (Li et al., 2009). Our results suggested that Hyp inhibited PHLPP2 expression, promoted AKT phosphorylation and GSK-3β inactivation to possess the liver protective effects *in vitro* and *in vivo*. In fact, Nrf2/AKT/GSK-3β/Fyn and AKT/Nrf2 signaling pathway are involved in the protection of natural products against xenobioticse-induced organ toxicity (Liu et al., 2018a; Liu et al., 2018b; Jin et al., 2019). In this present study, we also found that the AKT/GSK-3β/Nrf2 was involved in the protection of Hyp against oxidative stress in liver cells. Especially, we depicted the upstream signaling pathway, i.e. PHLPP2 participated in the protective mechanism. Our study provided data enriching the signaling pathway against oxidative stress.

However, our current study does have limitations; for example, we can’t exclude the functions of other PHLPP subtypes, such as PHLPP1 in participating this feedback loop. We also can’t explain how Hyp-induced PHLPP2 degradation occurred, the solely important function to sustain the stability of the feedback loop. In the present study, PHLPP2 siRNA was used to confirm the role of PHLPP2-AKT-GSK-3β signaling pathway in Hyp-mediated protection of the oxidative damaged L02 cells. In future study, PHLPP2 overexpressed L02 cells and PHLPP2 knockout mice would be efficient to confirm the function of PHLPP2-AKT-GSK-3β signaling pathway in Hyp-mediated protection against oxidative stress-induced liver injury.

## Conclusion

In conclusion, our current study revealed the protective effects of Hyp against oxidative stress-induced liver damage by regulation of the PHLPP2-AKT-GSK-3β signaling pathway. Moreover, our recent findings also suggested PHLPP2-AKT-GSK-3β kinase axis as a potential therapeutic target for liver damage. Future study will assess such Hyp protective effects against liver injury preclinically and clinically.

## Data Availability Statement

The raw data supporting the conclusions of this article will be made available by the authors, without undue reservation, to any qualified researcher.

## Ethics Statement

The animal protocol of this study was approved by the Institutional Animal Care and Use Committee (IACUC) of the Army Medical University (Chongqing, China) (Approval #201510003 and 201612010).

## Author Contributions

HX: Conceptualization, Methodology. RF: Data collection. CC: Writing-Original Draft. YC: Software. XW: Data Analysis. DD: Data collection and funding. XG: Validation, Writing—Review and Editing. JC: Supervision, Project administration. All authors contributed to the article and approved the submitted version.

## Funding

This work was supported in part by grants from the Chongqing Federation of Social Sciences Circles (grant number 2018PY79) and the Training Program of Clinical Medical Researcher of Army Medical University (grant number 2018XLC3072). The funders had no role in the design and conduct of this study, or collection and interpretation of the data, as well as no role in preparation and approval of the manuscript. This project was also supported by the grants from the National Natural Science Foundation of China (grant number. 81302867).

## Conflict of Interest

The authors declare that the research was conducted in the absence of any commercial or financial relationships that could be construed as a potential conflict of interest.
